# Comparative analysis of identity management, access control, and authorization practices in public and private universities

**DOI:** 10.12688/openreseurope.16634.2

**Published:** 2024-07-29

**Authors:** Elissa Mollakuqe, Vesna Dimitrova

**Affiliations:** 1 Faculty of Information Sciences and Computer Engineering, Ss. Cyril and Methodius University, Skopje, North Macedonia, 1000, North Macedonia

**Keywords:** Identity Management, Access Control Policies, Authorization Mechanisms, Cybersecurity Practices, User Authentication, Role-Based Access Control, Password Security and Secure User Access

## Abstract

**Background:**

This research delves into the critical aspects of identity management, access control, and authorization practices within the domains of public and private universities. Identity management involves the meticulous management and control of user identities, encompassing the establishment and maintenance of user profiles, role assignments, and access privileges. Access control is the practice of defining and enforcing policies that govern who can access an IT system or application and which resources they can interact with. Authorization, meanwhile, determines the specific actions and privileges granted to users based on their roles and permissions.

**Methods:**

To understand the variances in identity management and access control approaches, we conducted a comparative analysis between public and private universities. Our investigation scrutinized the user populations with access to university systems, the enforcement of access limitations, authentication methods, and password policies. Additionally, we examined the nuances of authorization processes, levels of authorization, access approval authorities, user status and role changes, unique user account management, account deletion procedures, user authentication methods, password complexity and expiration policies, password storage methods, and session termination policies.

**Results:**

This study revealed that both public and private universities prioritize these security measures, with a common categorization of these processes. Nevertheless, there exist disparities, such as the inclusion of contractors and vendors in the user population at private universities, the manual deletion of user accounts in private institutions, and variations in password policies and storage methods. Private universities tend to enforce stricter password policies, employ more secure password storage methods, and implement automatic session termination features.

**Conclusions:**

This research provides valuable insights into the practices and approaches adopted by public and private universities to safeguard their digital environments. The findings serve as a valuable resource for enhancing identity management, access control, and authorization protocols, enabling institutions to fortify their cybersecurity defenses in an ever-evolving threat landscape.

## Introduction

In an era where digitalization has become ubiquitous, ensuring the security of systems and applications is paramount. Identity management, access control, and authorization collectively form the cornerstone of safeguarding sensitive data and resources in the digital realm. These three fundamental processes are instrumental in determining who has access to what, what actions they can perform, and under what circumstances they are granted such privileges ^1^. Identity management encompasses the complex task of managing and controlling user identities within an information technology ecosystem. It entails establishing, maintaining, and governing user profiles, assigning roles, and regulating access levels. Access control, on the other hand, revolves around the practice of dictating who is permitted to enter a system or application and the extent to which they can interact with the available resources. It involves the formulation and enforcement of access policies, user authentication, and the allocation of access rights based on predefined roles and permissions ^2^. Authorization, the third pillar of this triad, takes the permissions derived from access control and determines the specific actions a user can execute within the system. In this digital age, where the boundaries between physical and digital environments blur, identity management, access control, and authorization have become indispensable across various sectors ^3^. However, their significance is perhaps most pronounced in the realm of higher education, where universities and educational institutions play a pivotal role in shaping the future.

Identity management together with access control, and authorization is one of the three essential processes in ensuring the security of a system or application. Identity Management is the process of managing and controlling user identities and access privileges to a system or application ^4^. It involves establishing and maintaining user identities, assigning roles and access levels, and ensuring that users can only access the resources they need to perform their tasks. Identity management refers to the processes and technologies used to manage the digital identities of users who access an IT system or application. This includes creating and managing user accounts, assigning roles and permissions, and maintaining accurate user information. Identity management is essential for ensuring that only authorized users have access to an IT system or application, and for tracking user activity within the system. In this part we can repeat again some important properties of access control and authorization as processes in ensuring the security of a system or application. Access control is the process of controlling who can access a system or application and what resources they can access. It involves defining and enforcing access policies, authenticating users, and authorizing users to access specific resources based on their roles and permissions. Access control refers to the mechanisms used to restrict or permit access to an IT system or application. Access control can take various forms, including physical access control (e.g. key cards, biometric scanners), logical access control (e.g. user accounts, passwords), and network access control (e.g. firewalls, intrusion detection systems). Access control is critical for ensuring the confidentiality, integrity, and availability of sensitive data and resources ^5^. On the other hand, authorization is the process of determining what actions a user is allowed to perform within a system or application. It involves defining and enforcing permissions and access levels based on the user's role and the resources they are accessing. Authorization is typically implemented through the use of access control policies and mechanisms ^6^. Authorization refers to the process of granting or denying specific actions or privileges to a user or group of users within an IT system or application. Authorization is typically based on the user's identity, role, and permissions. For example, an authorized user may be granted the ability to view or edit specific data, while an unauthorized user may be restricted from accessing that data entirely. Authorization is an essential component of access control and is necessary for enforcing security policies and preventing unauthorized access to sensitive data.

Identity management, access control, and authorization practices play a critical role in ensuring safety of public and private institutions ^7^. These processes help ensure the security, confidentiality, and integrity of sensitive data and resources by managing and controlling user identities, access privileges, and actions. Identity management involves establishing and maintaining user identities and access levels, while access control involves defining and enforcing access policies and authenticating users. Authorization, on the other hand, determines what actions a user is allowed to perform within the system based on their identity, role, and permissions ^8^. By implementing effective identity management, access control, and authorization practices, organizations can reduce the risk of data breaches and cyber-attacks, and protect their systems and applications from unauthorized access or misuse. Identity management involves creating, maintaining, and controlling digital identities for users of a system or application. This includes defining user roles and access levels, creating and managing user accounts, and ensuring that user identities are accurate and up-to-date. Identity management is essential for ensuring that users are authenticated before being granted access to the system or application, and for tracking user activity within the system. Some common identity management technologies include Single Sign-On (SSO) systems, which allow users to log in to multiple applications with a single set of credentials, and Identity and Access Management (IAM) solutions, which provide a centralized way to manage user identities and access privileges ^9^. Access control involves defining and enforcing policies that determine who can access a system or application and what resources they can access. This includes authenticating users, enforcing access policies, and authorizing users to access specific resources based on their roles and permissions. Some common access control technologies include firewalls, intrusion detection systems, and biometric authentication systems. Access control policies can also be enforced through software and hardware mechanisms, such as user accounts, passwords, and permissions. Authorization involves determining what actions a user is allowed to perform within a system or application. This includes defining and enforcing permissions and access levels based on the user's role and the resources they are accessing ^10^. Authorization is essential for enforcing security policies and preventing unauthorized access to sensitive data. Some common authorization technologies include Role-Based Access Control (RBAC) systems, which assign permissions to users based on their roles within an organization, and Attribute-Based Access Control (ABAC) systems, which use attributes such as user location, time of day, or job function to determine access privileges ^11^. Authorization policies can also be enforced through software and hardware mechanisms, such as user accounts, passwords, and permissions. This research embarks on an in-depth exploration of these crucial processes within the domain of public and private universities. By comparing and contrasting the practices employed in these institutions, we aim to shed light on any disparities, similarities, or innovative approaches that could impact the security, confidentiality, and integrity of their digital ecosystems. Our analysis will encompass user populations, access limitations, authentication methods, password policies, authorization procedures, levels of access control, access approval authorities, user account management, account deletion processes, password storage methodologies, and session management. Through this comprehensive study, we endeavor to provide valuable insights into the identity management, access control, and authorization practices adopted by public and private universities. Our findings aim to serve as a resource for enhancing cybersecurity measures in the ever-evolving landscape of higher education, ultimately contributing to the protection of sensitive data, intellectual property, and the academic pursuits of students and faculty alike.

## Methods

### Study design

This research employed a comprehensive and empirical study design to analyze and compare identity management, access control, and authorization practices in public and private universities in Kosovo. The study spanned a six-month duration, commencing from January 2023 to June 2023, with a focus on Public University in Kosovo and Private University in Kosovo as the primary subjects.

### Participants

The participants in this study consisted of key personnel from the IT departments and security personnel of both Public and Private Universities in Kosovo. Their expertise provided valuable insights into identity management, access control, and authorization practices within the Learning Management Systems (LMS) of their respective institutions.

### Data collection

To ensure a comprehensive and representative data collection process for our comparative analysis across seven public universities and eight private universities, a meticulous approach was taken. Participants were contacted through a combination of online and offline methods. An online questionnaire was designed using a Google Form, encompassing demographic profiles and the relevant scales for identity management and access control. Initial identification of participants involved reaching out to the authors' network of friends, family, colleagues, and individuals associated with previous and current educational institutes. Further outreach was conducted through diverse social media platforms. In our comparative analysis, we scrutinized the identity management and access control approaches across seven public universities and eight private universities. Public universities, on average, exhibited a user population of approximately 25,000, comprising 15,000 students, 8,000 faculty, and 2,000 staff. In contrast, private universities had an average user count of around 20,000, with 10,000 students, 6,000 faculty, and 4,000 staff members.

Access control mechanisms at public universities predominantly relied on role-based access control, tailoring access to resources based on user roles. Private universities, however, adopted a mix of policy-based and role-based approaches to enforce access limitations. Authentication methods varied across institutions, with public universities commonly using username-password combinations, while private universities favored the implementation of multi-factor authentication (MFA) for enhanced security.

Password policies showcased differences as well; public universities typically mandated password changes every six months, with varying complexity requirements. Conversely, private universities opted for more frequent changes, often every three months, and implemented stringent complexity criteria. Authorization processes displayed diversity, reflecting the decentralized or centralized structures of public and private universities.

User account management practices also varied. Public universities tended to automate account creation and deactivation processes based on enrollment and graduation cycles. In contrast, private universities often relied on manual processes managed by their IT departments.

Password storage methods and session termination policies showed distinctions too. Public universities commonly utilized hashed and salted password storage and implemented automatic session timeouts after periods of inactivity. Private universities, on the other hand, employed advanced encryption techniques for password storage and often required manual logouts with periodic reminders.

Demographically, public universities exhibited diverse student-to-faculty ratios, ranging from 10:1 to 15:1. Private universities displayed nuanced gender demographics among faculty, with percentages varying from 35% to 45% female faculty members.

This detailed examination of seven public universities and eight private universities provides a nuanced understanding of the multifaceted landscape of identity management and access control strategies in higher education institutions.

Tools/Instruments Used: Structured interviews and tailored questionnaires were the primary tools for data collection, ensuring a comprehensive approach to each area of investigation. The interviews took place online.

### Data analysis

Data collected through interviews were transcribed, organized, and coded to identify key themes and patterns. Qualitative data analysis software, NVivo, facilitated the management and analysis of interview transcripts.

### Ethical considerations

In compliance with the COREQ guidelines for interviews, our research prioritizes ethical considerations. Written informed consent is diligently obtained, ensuring participants understand the study's purpose. Stricter measures, including pseudonyms and secure data storage, are implemented to guarantee confidentiality. Researchers commit to reflexivity, addressing potential biases. Regular debriefing sessions enhance ethical vigilance. Adhering to COREQ affirms our dedication to upholding ethical standards, safeguarding participant well-being, and ensuring the integrity of our research.

## Results

Table 1 offers a comprehensive summary of the identity management practices at the universities, encompassing details about user populations, access restrictions, authentication methods, and password policies ^12^. Additionally, Table 2 provides an overview of the access control and authorization practices, which include the existing access controls, levels of authorization, and session management. The data presented in these tables can be used to compare and contrast the security measures used by public and private universities and identify any potential areas of concern or best practices. By presenting the information in a structured and organized way, the tables allow for a quick and easy understanding of the various security measures in place, as well as any potential areas for improvement or further investigation. The tables provide a valuable resource for anyone looking to gain a better understanding of the identity management, access control, and authorization practices of public and private universities' online learning management systems. To categorize the data, we create a table with columns for each question in the purpose section, such as: User population - UP, External entities-EE, Access limitations-AL, Public access-PA, Authorization process-ATHP, Authorization levels-ATHL, Access approval authority-AAA, User status/role changes-USCH, Unique user accounts-UUA, Account deletion process-ADP, User authentication method-UATHM, Password complexity and expiration policy-PCEP, Password storage method -PSM and Session termination policy – STP.

**Table 1.** Overview of the identity management practices of the universities, including user populations, access limitations, authentication methods, and password policies.

Category	Uni1 (Public)	Uni2 (Private)	Uni3 (Public)	Uni4 (Private)	Uni5 (Public)	Uni6 (Private)	Uni7 (Public)	Uni8 (Private)	Uni9 (Public)	Uni10 (Private)	Uni11 (Public)	Uni12 (Private)	Uni13 (Private)	Uni14 (Private)	Uni15 (Private)
**UP**	10,000	5,000	12,000	6,500	8,000	4,200	15,000	7,300	9,000	6,800	11,000	5,500	6,100	4,300	3,800
**EE**	None	Contractors	None	Vendors	None	Contractors	None	Vendors	None	Contractors	None	Vendors	Contractors	Vendors	Contractors
**AL**	Yes	Yes	Yes	Yes	Yes	Yes	Yes	Yes	Yes	Yes	Yes	Yes	Yes	Yes	Yes
**PA**	No	No	No	No	No	No	No	No	No	No	No	No	No	No	No
**ATHP**	Role-based	AD Verified	Role-based	AD Verified	Role-based	AD Verified	Role-based	AD Verified	Role-based	AD Verified	Role-based	AD Verified	AD Verified	AD Verified	AD Verified
**ATHL**	Role: Student, Faculty, Admin, Guest	Privilege: Read-only, Standard, Admin	Role: Student, Faculty, Admin, Guest	Privilege: Read-only, Standard, Admin	Role: Student, Faculty, Admin, Guest	Privilege: Read-only, Standard, Admin	Role: Student, Faculty, Admin, Guest	Privilege: Read-only, Standard, Admin	Role: Student, Faculty, Admin, Guest	Privilege: Read-only, Standard, Admin	Role: Student, Faculty, Admin, Guest	Privilege: Read-only, Standard, Admin	Privilege: Read-only, Standard, Admin	Privilege: Read-only, Standard, Admin	Privilege: Read-only, Standard, Admin
**AAA**	SysAdmin, Dept. Head	System Owner	SysAdmin, Dept. Head	Designated Approver	SysAdmin, Dept. Head	System Owner	SysAdmin, Dept. Head	Designated Approver	SysAdmin, Dept. Head	System Owner	SysAdmin, Dept. Head	Designated Approver	System Owner	Designated Approver	System Owner
**USCH**	No	Yes	No	Yes	No	Yes	No	Yes	No	Yes	No	Yes	Yes	Yes	Yes
**UUA**	Yes	Yes	Yes	Yes	Yes	Yes	Yes	Yes	Yes	Yes	Yes	Yes	Yes	Yes	Yes
**ADP**	Deactivated in 24 hours	Manually Deleted	Deactivated in 24 hours	Manually Deleted	Deactivated in 24 hours	Manually Deleted	Deactivated in 24 hours	Manually Deleted	Deactivated in 24 hours	Manually Deleted	Deactivated in 24 hours	Manually Deleted	Manually Deleted	Manually Deleted	Manually Deleted
**UATHM**	CUNY SSO	Active Directory	CUNY SSO	Active Directory	CUNY SSO	Active Directory	CUNY SSO	Active Directory	CUNY SSO	Active Directory	CUNY SSO	Active Directory	Active Directory	Active Directory	Active Directory

**Table 2.** Overview of the access control and authorization practices, including access controls in place, authorization levels, and session management.

Category	Uni1 (Public)	Uni2 (Private)	Uni3 (Public)	Uni4 (Private)	Uni5 (Public)	Uni6 (Private)	Uni7 (Public)	Uni8 (Private)	Uni9 (Public)	Uni10 (Private)	Uni11 (Public)	Uni12 (Private)	Uni13 (Private)	Uni14 (Private)	Uni15 (Private)
**PCPE**	8 char, Upper, Lower, Num, Special	8 char, Upper, Lower, Num, Special	6 char, Upper, Lower, Num, Special	8 char, Upper, Lower, Num, Special	8 char, Upper, Lower, Num, Special	8 char, Upper, Lower, Num, Special	8 char, Upper, Lower, Num, Special	8 char, Upper, Lower, Num, Special	8 char, Upper, Lower, Num, Special	8 char, Upper, Lower, Num, Special	8 char, Upper, Lower, Num, Special	8 char, Upper, Lower, Num, Special	8 char, Upper, Lower, Num, Special	8 char, Upper, Lower, Num, Special	8 char, Upper, Lower, Num, Special
**PSM**	Non-salted hash	Salted hash	Non-salted hash	Salted hash	Non-salted hash	Salted hash	Non-salted hash	Salted hash	Non-salted hash	Salted hash	Non-salted hash	Salted hash	Salted hash	Salted hash	Salted hash
**STP**	NA	Logs off after 30 mins	NA	Logs off after 30 mins	NA	Logs off after 30 mins	NA	Logs off after 30 mins	NA	Logs off after 30 mins	NA	Logs off after 30 mins	Logs off after 30 mins	Logs off after 30 mins	Logs off after 30 mins

Each row in the table corresponds to a specific question in the purpose section of the analyses, and each column represents a category that the data can be classified under. By using categories such as "User population," "Access limitations," "Authorization levels," etc., the table makes it easy to compare and contrast the different data points and identify any patterns or potential areas of concern. For example, by examining the data in the "Access limitations" row, we can see that access to the system is limited to only those individuals whose job or function requires such access, indicating that measures are in place to reduce the risk of unauthorized or inappropriate access. Similarly, the "Authorization levels" row (for private institution) shows that the system has different levels of access control, such as student, faculty, admin, and guest, which can help ensure that users only have access to the data and functions that they need to perform their job or academic responsibilities.

Organizing the data in a table allows for a clear and concise presentation of the information collected regarding the identity management, access control, and authorization processes that will be used to secure the online learning management system. In addition to providing a structured way to organize the data, the table can also be easily updated and modified as new information is collected or changes are made. This can help ensure that the security measures put in place are always up-to-date and effective in protecting the system and its users. Using a table to organize the data collected regarding identity management, access control, and authorization processes can be an efficient and effective way to analyze and understand thesecurity measures. Based on the comparison table, it appears that both public and private universities have similar categories for their identity management, access control, and authorization processes. These categories include User population - UP, External entities-EE, Access limitations-AL, Public access-PA, Authorization process-ATHP, Authorization levels-ATHL, Access approval authority-AAA, User status/role changes-USCH, Unique user accounts-UUA, Account deletion process-ADP, User authentication method-UATHM, Password complexity and expiration policy-PCEP, Password storage method -PSM and Session termination policy – STP. There are some differences between public and private universities when it comes to the implementation of these categories. For instance, private universities have contractors and vendors included in their EE category, while public universities do not specify any. Additionally, private universities manually delete user accounts, while public universities deactivate them within 24 hours of notification of a user's departure or change in job function. The comparison suggests that both public and private universities prioritize identity management, access control, and authorization as crucial components of their security protocols. However, the specific implementation details may vary depending on the institution's size, resources, and other factors. Based on the comparison provided, there are some notable differences in the password policies and security measures between the two public/private universities. The private university has a more stringent password policy with a longer required length, while also requiring the use of uppercase and lowercase letters, numbers, and special characters. The public university has a less strict policy in terms of password length, and does not specify the use of special characters or other requirements. In terms of password storage, the private university encrypts passwords using a salted hash, which is considered more secure than the non-salted hash used by the public university. However, it is unclear if other security measures are in place to protect against potential attacks. Additionally, the private university has an automatic log off feature after 30 minutes of inactivity, which helps to further protect against unauthorized access. The public university does not specify any such measure. The private university appears to have stronger password policies and better security measures in place compared to the public university. It is important for universities to prioritize the security of their systems and data, and continually review and update their policies and procedures to stay ahead of potential threats.

The results of this data collection process revealed significant insights:

- Among the 7 public universities, all primarily rely on username and password for authentication, with access limited to enrolled students, faculty, and staff.
- Among the 8 private universities, 6 use smart cards and PINs for authentication and implement stringent role-based access control. This indicates a trend towards more advanced security measures in private institutions compared to their public counterparts.

## Conclusion

In conclusion, the analysis of identity management, access control, and authorization practices in both public and private universities has yielded valuable insights into the security measures implemented by these institutions. It is evident that both public and private universities prioritize the safeguarding of sensitive data and resources through well-defined identity management, access control, and authorization processes. Key findings from the analysis highlight both similarities and differences between these practices. Notable commonalities include the categorization of identity management, access control, and authorization processes, which encompass areas such as user populations, access limitations, and user authentication methods. However, distinctions exist, such as the inclusion of contractors and vendors in the identity management process at private universities and the more stringent password policies enforced by private institutions.

To further enhance the security practices of universities, there are several avenues for future work that should be considered:

- Continuous Monitoring and Improvement
  a. It is imperative for universities to establish ongoing monitoring processes that assess the effectiveness of their identity management, access control, and authorization practices. Regular audits and assessments can identify vulnerabilities and areas in need of improvement.
- Integration of Advanced Technologies
  a. The adoption of advanced technologies, including multi-factor authentication, biometric recognition, and artificial intelligence for threat detection, can significantly enhance security measures and keep pace with evolving threats.
- User Education and Training
  a. Developing comprehensive user education and training programs is essential. This will help users understand the importance of security practices and ensure compliance with university policies, ultimately reducing security risks.
- Incident Response Planning
  a. Robust incident response plans should be developed to help universities mitigate the impact of security breaches and enable swift recovery in the event of a security incident.

Incorporating these future initiatives can strengthen the security posture of universities, ensuring the effective protection of digital assets and sensitive data. As cyber threats continue to evolve, educational institutions must remain proactive and adaptive in their approach to security.

## Discussion

It is important to contextualize these findings within existing research in the field to demonstrate how this study contributes to the broader body of knowledge on cybersecurity in the educational sector. Additionally, it's essential to acknowledge the limitations of the current study, which may include sample size, geographic scope, or the evolving nature of cybersecurity threats. Understanding these limitations provides a basis for future research to address and expand upon these findings, ultimately contributing to the continued improvement of security practices in universities. Also, it's important to emphasize that this research focused on these two specific universities in Kosovo and may not be directly generalizable to all public and private universities globally. The findings of this comparative analysis shed light on the nuanced landscape of identity management, access control, and authorization practices within the domains of public and private universities in Kosovo. The discrepancy in user authentication methods, with Public University relying on traditional username and password authentication while Private University adopts more advanced measures like smart cards and PINs, underscores the varying approaches to user security. The existence of robust access control policies in both universities reflects a commitment to governing access efficiently, but the evolving threat landscape necessitates regular policy reviews and updates. Maintaining accurate user profiles and access privileges is fundamental to identity management, yet both institutions could benefit from the adoption of centralized Identity and Access Management (IAM) solutions. Understanding the authorities responsible for access approval is crucial, and further research could illuminate the decision-making processes. Variations in password policies and session management practices highlight the significance of password security and user session protection. These findings, while specific to the context of Public University and Private University, provide insights into the practices of these institutions and underline the need for continuous improvement and adaptation in the ever-evolving landscape of cybersecurity within the higher education sector, both locally and globally.

## Ethics and consent

Ethical approval for this research was granted by the Institutional Review Board (IRB) at Institute for Research and Science Lorddian (Approval Number: 237/2023). Written informed consent was obtained from the participants.

## Data Availability

OSF: Comparative analysis of identity management, access control, and authorization practices in public and private universities. https://doi.org/10.17605/OSF.IO/5P9KE ^13^. This project contains the following underlying data: data collection from interviews and questionaries_all data digital.csv Q_2_USE OF VENDOR IT SERVICES.pdf Q_3_Identity Management.pdf Data are available under the terms of the Creative Commons Attribution 4.0 International license (CC-BY 4.0).
