# Supplementary material for: Comparative analysis of identity management, access control, and authorization practices in public and private universities
Source: Open Res Eur. 2024 Jul 29;4:23. Originally published 2024 Feb 9. [Version 2] doi: 10.12688/openreseurope.16634.2 (PMC11294802; doi:10.12688/openreseurope.16634.2)
Supplement: Peer review history

# Reviewer report: Reviewer response for version 2

**DOI:** 10.21956/openreseurope.19737.r42582

**Published:** 2024-08-01

**Authors:** Wanpeng Li

**Affiliations:** 1 Department of Computing Science, University of Aberdeen, Aberdeen, Scotland, UK

The authors conducted a comparative analysis of identity management, access control, and authorization practices within the Learning Management Systems of 15 universities in Kosovo. Overall, the paper is very insightful and beneficial for understanding identity management practices in Kosovo. And the authors addressed the issues in the first version.

Is the study design appropriate and does the work have academic merit?

Yes

Is the work clearly and accurately presented and does it cite the current literature?

Yes

If applicable, is the statistical analysis and its interpretation appropriate?

Partly

Are all the source data underlying the results available to ensure full reproducibility?

No

Are the conclusions drawn adequately supported by the results?

Partly

Are sufficient details of methods and analysis provided to allow replication by others?

No

Reviewer Expertise:

Identity management, Web security

I confirm that I have read this submission and believe that I have an appropriate level of expertise to confirm that it is of an acceptable scientific standard.

# Reviewer report: Reviewer response for version 1

**DOI:** 10.21956/openreseurope.17960.r41627

**Published:** 2024-07-09

**Authors:** Wanpeng Li

**Affiliations:** 1 Department of Computing Science, University of Aberdeen, Aberdeen, Scotland, UK

The authors conducted a comparative analysis of identity management, access control, and authorization practices within the Learning Management Systems of 15 universities in Kosovo. Overall, the paper is very insightful and beneficial for understanding identity management practices in Kosovo. However, there are a few issues that the authors need to address:

 1. The data provided in Table 1 only categorizes universities as public or private. More detailed data could enhance the analysis. If privacy is a concern, pseudonyms could be used to describe the universities included in the study.

 2. In the Results section, the authors state, "by examining the data in the 'Access limitations' row, we can see that access to the system is limited to only those individuals whose job or function requires such access, indicating that measures are in place to reduce the risk of unauthorized or inappropriate access." However, this conclusion is unclear from Table 1, as only the 'Yes' value is provided.

 3. Table 2 lacks detailed information. Additional data should be provided, such as how many universities use salted hash and how many automatically log off users after 30 minutes.

 4. In the last paragraph of the Results section, the authors claim that:

 - Public universities primarily rely on username and password for authentication, with access limited to enrolled students, faculty, and staff.

 - Private universities use smart cards and PINs for authentication and implement stringent role-based access control.

 However, no supplementary data is provided to support these claims. It is unclear how many public and private universities fall into these categories.

Is the study design appropriate and does the work have academic merit?

Yes

Is the work clearly and accurately presented and does it cite the current literature?

Yes

If applicable, is the statistical analysis and its interpretation appropriate?

Partly

Are all the source data underlying the results available to ensure full reproducibility?

No

Are the conclusions drawn adequately supported by the results?

Partly

Are sufficient details of methods and analysis provided to allow replication by others?

No

Reviewer Expertise:

Identity management, Web security

I confirm that I have read this submission and believe that I have an appropriate level of expertise to confirm that it is of an acceptable scientific standard, however I have significant reservations, as outlined above.

# Reviewer report: Reviewer response for version 1

**DOI:** 10.21956/openreseurope.17960.r38517

**Published:** 2024-03-14

**Authors:** Zonara Telaku

**Affiliations:** 1 UBT Kosova, Prishtina, Kosovo (Serbia and Montenegro)

This research investigates identity management, access control, and authorization practices in public and private universities in Kosovo. Through a comparative analysis, the study examines user populations, access limitations, authentication methods, password policies, authorization processes, and session management. Key findings reveal both similarities and differences between public and private universities, highlighting the importance of continuous improvement in security practices. Recommendations for future work include expanding the sample size, addressing study limitations, integrating stakeholder perspectives, and ensuring scientific soundness. A comprehensive and empirical study design is employed, involving key personnel from IT departments and security personnel in public and private universities in Kosovo. Data collection methods include structured interviews and tailored questionnaires, with data analysis facilitated by qualitative data analysis software. Ethical considerations are meticulously addressed to ensure research integrity. The results section presents detailed findings from the comparative analysis, organized into tables for easy reference. It highlights similarities and differences in identity management, access control, and authorization practices between public and private universities. Notable findings include variations in authentication methods, password policies, and session management practices.

Is the study design appropriate and does the work have academic merit?

Yes

Is the work clearly and accurately presented and does it cite the current literature?

Yes

If applicable, is the statistical analysis and its interpretation appropriate?

Partly

Are all the source data underlying the results available to ensure full reproducibility?

Yes

Are the conclusions drawn adequately supported by the results?

Yes

Are sufficient details of methods and analysis provided to allow replication by others?

Yes

Reviewer Expertise:

Computer Science

I confirm that I have read this submission and believe that I have an appropriate level of expertise to confirm that it is of an acceptable scientific standard.
